# Serum neurofilament light chain levels are associated with all-cause mortality in the general US population

**DOI:** 10.1007/s00415-023-11739-6

**Published:** 2023-04-21

**Authors:** Stefano Ciardullo, Emanuele Muraca, Eleonora Bianconi, Celeste Ronchetti, Rosa Cannistraci, Laura Rossi, Silvia Perra, Francesca Zerbini, Gianluca Perseghin

**Affiliations:** 1 Department of Medicine and Rehabilitation, Policlinico di Monza, Via Modigliani 10, 20900 Monza, MB Italy; 2 Department of Medicine and Surgery, University of Milano Bicocca, Milan, Italy

**Keywords:** Serum neurofilament, Mortality, Epidemiology, Neurologic disorders

## Abstract

**Introduction:**

Serum neurofilament light chain (sNfL) levels are biomarkers of neuro-axonal injury in multiple neurological diseases. Little is known on their potential role as prognostic markers in people without known neurological conditions.

**Objective:**

The aim of this study is to evaluate the association between sNfL levels and all-cause mortality in a general population setting.

**Methods:**

sNfL levels were measured in 2071 people aged 25–75 years from the general US population that participated in the 2013–2014 cycles of the National Health and Nutrition Examination Survey (NHANES). Cognitive function was evaluated in a subset of participants aged 60–75 years using the Consortium to Establish a Registry for Alzheimer’s Disease-Word Learning test, the Animal Fluency test and the Digit Symbol Substitution test. We applied Cox proportional hazard models adjusted for several potential confounders to evaluate the association between sNfL and all-cause mortality through December 2019 by linking NHANES data with data from the National Death Index.

**Results:**

In a cross-sectional analysis, higher sNfL levels were associated with worse performance in all three cognitive function tests. Over a median follow-up of 6.1 years, 85 participants died. In a multivariable model adjusted for age, sex, race-ethnicity, diabetes, chronic kidney disease, harmful alcohol consumption, cigarette smoke and prevalent cardiovascular disease, higher sNfL levels were significantly and positively associated with all-cause mortality (HR per unit increase in log-transformed sNfL: 2.46, 95% CI 1.77–3.43, *p* < 0.001). Results were robust when analyses were stratified according to age, sex, body mass index and kidney function.

**Conclusion:**

We found a positive association between sNfL levels and mortality in the general US population. Further studies are needed to understand the biological mechanisms underlying this association.

## Introduction

Neurofilaments are a type of neuron-specific intermediate filaments that are made up of light, medium, and heavy chains [1]. They are the primary proteins of the neural cytoskeleton and are released into the external environment when neuro-axonal damage occurs, which makes them potential biomarkers for various neurological diseases [2, 3]. The neurofilament light chain (NfL) is particularly promising as a biomarker due to its high solubility [4]. In the past, studies of neurofilaments were limited to cerebrospinal fluid (CSF), because detection systems were not sensitive enough to detect the lower levels of NfL in peripheral blood, which limited its clinical applications. However, obtaining CSF requires an invasive procedure (lumbar puncture), which is usually performed only for diagnostic purposes [5]. Recent studies have shown that the levels of NfL in the CSF and blood (sNfL) are highly correlated, which has increased interest in using sNfL as a biomarker for various neurological disorders in large-scale studies [6].

Beside its use for diagnostic purposes, sNfL has shown promise as a prognostic biomarker. Previous studies that measured sNFL levels in a general population setting showed that they are positively associated with incident all-cause dementia, Alzheimer’s disease and cognitive decline [7, 8]. More recently, it has also been proposed as a predictor of all-cause mortality in the elderly [9], as well as in middle-aged and African American individuals without known neurologic disorders [10, 11]. Nonetheless, whether sNfL are associated with all-cause mortality in an unselected population of adults from the general population is currently unknown.

In this context, the aim of the present study is to evaluate the association between sNFL and all-cause mortality in the general multiethnic population of the United States. To achieve this goal, we conducted a cohort study based on data obtained during the 2013–2014 cycle of the National Health and Nutrition Examination Survey (NHANES), which were linked to the National Death Index, providing follow-up through December 31, 2019.

## Methods

This is an analysis of data from the 2013–2014 cycles of NHANES, which is conducted in the United States by the National Center for Health Statistics (NCHS).

All data analyzed in the present study are freely available online at the CDC website (https://wwwn.cdc.gov/nchs/nhanes/continuousnhanes/default.aspx?BeginYear=2013).

It is an ongoing cross-sectional complex survey aimed at including individuals representative of the general, non-institutionalized population of all ages. To this end, it applies a stratified, multistage, clustered probability sampling design with oversampling of non-Hispanic black and Hispanic persons, people with low income and older adults. The survey consists of a structured interview conducted in the home, followed by a standardized health examination that includes a physical examination as well as laboratory tests. Full methodology of data collection is available elsewhere [12]. The original survey was approved by the Centers for Disease Control and Prevention Research Ethics Review Board and written informed consent was obtained from all adult participants. The present analysis was deemed exempt by the Institutional Review Board at our institution, as the dataset used in the analysis was completely de-identified.

### Laboratory tests and clinical data

Participants self-reported age, sex, race-ethnicity (categorized as non-Hispanic white, non-Hispanic black, Hispanic or other), education, smoking status and previous medical history. Body measurements including height (cm), weight (kg) and waist circumference (cm) were ascertained during the mobile examination center visit; BMI was calculated as weight in kilograms divided by height in meters squared.

Alcohol consumption was estimated based on self-reported data on the amount and frequency of alcohol use within the previous year. It was considered significant if > 1 drink per day for women and > 2 drinks per day for men on average [13].

Diabetes was defined in accordance with the American Diabetes Association criteria [14, 15]. Laboratory methods for measurements of HbA1c, glucose, lipid profile, platelet count and creatinine are reported in detail elsewhere [16]. Estimated glomerular filtration rate (eGFR) was computed according to the Chronic Kidney Disease Epidemiology Collaboration (CKD-EPI) equation and CKD was defined as an eGFR < 60 mL/min/1.73 m^2^. Diagnoses of heart failure (HF), coronary artery disease (CAD) and stroke were based on self-report. Patients reporting either CAD or stroke were classified as having cardiovascular disease (CVD).

### Serum neurofilament light chain measurement

Sera from stored surplus specimens from participants aged 20–75 years in a half-sample from NHANES 2013–2014, who consented testing their samples for future research and had stored surplus or pristine serum samples were eligible. The biobanked specimens are aliquoted and frozen at −20 °C until they are shipped weekly to the CDC’s central laboratory and where they are stored at −80 °C. Quality control and quality assurance protocols for NHANES mobile centers are Clinical Laboratory Improvement Act (CLIA) compliant.

Measurements were performed using a highly sensitive NfL immunoassay that utilizes acridinium ester (AE) chemiluminescence and paramagnetic particles and was run on an existing, high-throughput, automated platform (Attelica).

Initially, the sample is incubated with acridinium-ester (AE) labeled antibodies, which bind to the NfL antigen. Following this step, paramagnetic particles (PMP) coated with capture antibody are added to the sample, forming complexes of antigen bound to AE-labeled antibodies and PMP. Unbound AE-labeled antibodies are then separated and removed, following which acid and base are added to initiate chemiluminescence and light emission is measured. All steps were performed on the fully automated Attelica immunoassay system. The lower limit of quantification (LLOQ) of the assay is 3.9 pg/mL, which was determined by replicate testing (*n* = 44) of low concentration NfL samples. The lower limit of quantification (LLOQ) was defined as the concentration at which the coefficient of variation (CV) was less than or equal to 20%. For analytes with analytic results below the LLOQ (*n* = 36 participants), an imputed fill value was placed in the analyte results field equal to the LLOQ divided by the √2. No participants had values above the upper limit of quantification (500 pg/mL).

### Cognitive function

Three cognitive function tests were performed in a subgroup of participants aged 60 years or older. The Consortium to Establish a Registry for Alzheimer’s Disease (CERAD W-L) assesses immediate and delayed learning ability for new verbal information [17, 18]. The CERAD W-L consists of three consecutive learning trials and a delayed recall. For the three learning trials, participants were instructed to read aloud ten unrelated words. Immediately following the presentation of the words, participants recalled as many words as possible. The delayed recall occurred approximately 10 min after the start of the word learning trials. The maximum score on each trial is 10; the maximum score for the total word list is 40 (sum of the three trials plus the delayed recall).

The Animal Fluency Test examines verbal category fluency, a component of executive function, as well as other functions such as semantic memory and processing speed [19]. Participants were asked to name as many animals as possible in 1 min with a point given for each named animal.

The Digit Symbol Substitution Test (DSST) is a global measure of brain health, relies on processing speed, visual scanning, sustained attention, and short-term memory [20]. The test is conducted using a paper form with a key at the top containing nine numbers paired with distinct symbols. Participants had 2 min to copy the corresponding symbols in the 133 boxes that adjoin the numbers.

### All-cause mortality

Mortality data from death certificates from the National Death Index were linked to NHANES based on the participant sequence number available on both datasets. The present analysis is based on the public-use linked mortality files for NHANES 2013–2014, which include follow-up time and cause of death for adult participants through 31 December 2019. Due to relatively low statistical power, we limited our analysis to all-cause mortality. More information on the linkage method and analytic guidelines are available on the specific webpage of the National Center for Health Statistics [32].

### Statistical analysis

All analyses were conducted using Stata version 16 (StataCorp, College Station, TX), accounting for the complex survey design of NHANES. We used appropriate weighting for each analysis, as suggested by the NCHS to obtain estimates that were generalizable to the US population aged 20–75 years. Data are expressed as weighted proportions (Standard Error (SE)) for categorical variables and as weighted means (SE) for continuous variables. Participants’ characteristics according to sNfL levels were compared using linear regression for continuous variables and the design-adjusted Rao-Scott chi-square test for categorical variables.

Trends in the performance in the cognitive function scores across quartiles of sNfL levels were assessed using multivariable linear regression. We modeled the specific score as a dependent variable and the sNfL quartiles as a continuous independent variable. Trends were adjusted for age, sex, BMI and education.

Cox proportional hazard models were applied to evaluate the association between sNfL and all-cause mortality, after adjustment for potential confounders. We verified the proportional hazard assumption by using log–log plots and by testing for interaction by log(time). The following potential confounders were included in the model: age, sex, race-ethnicity, BMI, education, cigarette smoke, CKD, diabetes, prevalent CVD and alcohol consumption. Choice of confounders was based on classical risk factors for overall mortality and on the association between sNfL levels and aging, reduced kidney function and diabetes [21]. sNfL levels were included as a log-transformed continuous variable. A two-tailed value of *p* < 0.05 was considered statistically significant.

## Results

### Features of the study population

Among a total of 5262 participants aged 20–75 years, 5106 attended a mobile examination center visit. We excluded 3035 individuals without an available sNfL measurement, leading to a population of 2071. Data on mortality were available in all participants.

**Fig. 1:**
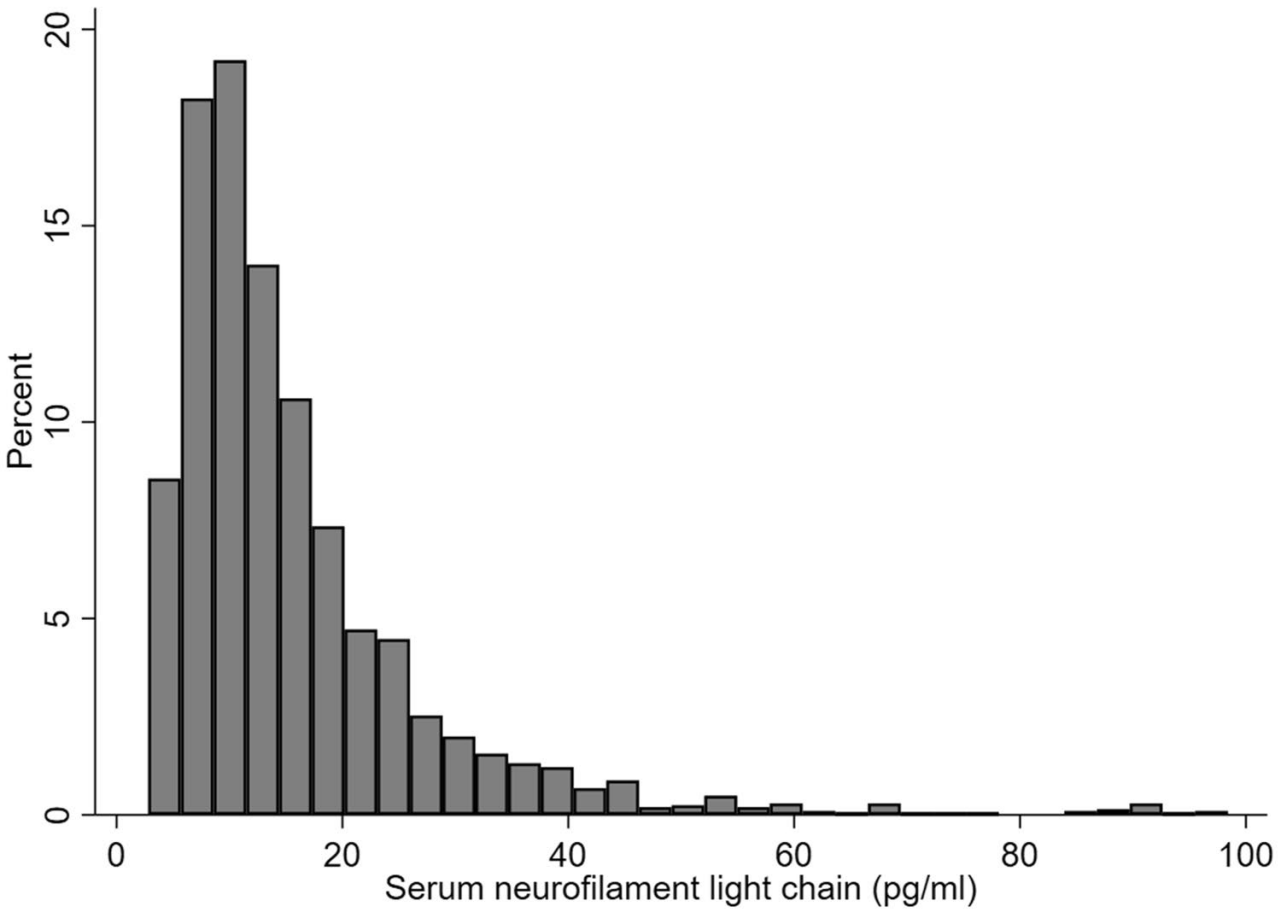
Distribution of serum neurofilament light chain (sNfL) levels in adult participants in the 2013–2014 National Health and Nutrition Examination Survey

**Fig. 2:**
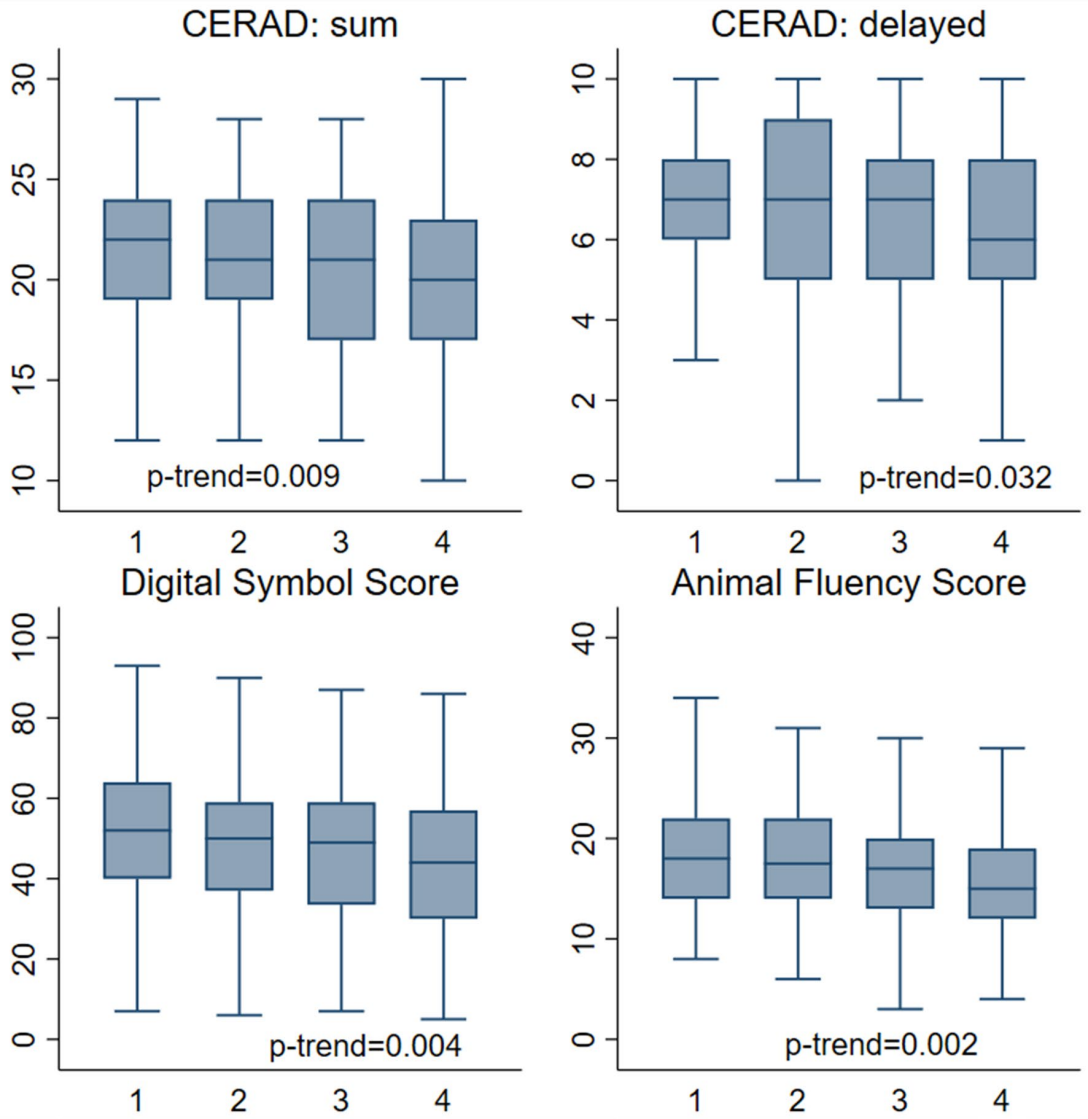
Cognitive assessment scores by quartiles of serum neurofilament light chain (sNfL) levels among adults age 60–75 years. *p*-values are adjusted for age, sex, body mass index and educational status. *CERAD* Consortium to Establish a Registry for Alzheimer’s disease Word Learning subtest

Overall distribution of sNfL levels in the studied population is depicted in Fig. 1. As shown, since the distribution was rightly skewed, sNfL levels were log-transformed before performing multivariate analysis. Clinical and biochemical features of the entire population divided by quartiles of sNfL are shown in Table 1. Participants with higher sNfL levels were older, more frequently male of non-Hispanic white ethnicity, with a lower proportion of never-smokers.They also had lower eGFR. Most comorbid conditions were more prevalent in quartile 4 compared with quartile 1, including diabetes, CVD and stroke. The association between sNFL levels and cognitive performance was evaluated in a subgroup of 521 participants aged 60–75 years with available data on both variables. Mean values on the CERAD, animal fluency and digit symbol scores across quartiles of sNfL levels are shown in Fig. 2. For all tests, a progressive decrease in the participants score was identified with increasing sNfL levels. This trend remained significant after adjustment for age, sex, BMI and educational status.

**Table 1:** Demographic, anthropometric and clinical characteristics of participants in the 2013–2014 National Health and Nutrition Examination Survey according to quartiles of serum neurofilament light chain (sNfL) levels

	sNfL quartiles, pg/mL				*p*-Trend
	2.8–8.2	8.3–12.3	12.4–19.1	≥ 19.2	
Age category (years)					< 0.001
20–34	266 (56.9)	143 (30.2)	88 (19.6)	44 (10.6)	
35–44	152 (27.3)	119 (24.0)	65 (14.9)	71 (13.9)	
45–54	73 (12.0)	122 (22.3)	114 (24.0)	74 (17.1)	
55–64	32 (3.7)	100 (17.3)	145 (24.5)	143 (29.6)	
65–75	1 (0.1)	37 (6.3)	99 (17.0)	182 (28.9)	
Sex					0.002
Male	215 (42.5)	254 (50.2)	251 (48.5)	270 (54.2)	
Female	309 (57.5)	267 (49.8)	260 (51.5)	244 (45.8)	
Race-ethnicity (%)					0.001
Non-Hispanic white	196 (54.0)	212 (62.7)	248 (71.9)	254 (72.5)	
Hispanic	146 (22.5)	126 (15.3)	110 (12.5)	105 (10.3)	
Non-Hispanic black	94 (14.0)	110 (14.4)	70 (8.1)	99 (11.4)	
Non-Hispanic Asian	67 (6.0)	65 (5.9)	72 (6.1)	43 (3.5)	
Other	21 (3.6)	8 (1.7)	11 (1.4)	13 (2.3)	
Education					0.137
High school or less	212 (36.1)	227 (37.2)	200 (33.5)	242 (36.3)	
Some college	176 (34.7)	149 (30.4)	155 (30.8)	172 (38.4)	
College or above	135 (29.3)	144 (32.4)	156 (35.8)	99 (25.3)	
Cigarette smoke					0.023
Never	329 (64.1)	309 (58.5)	266 (52.3)	250 (49.7)	
Former	83 (15.7)	108 (21.8)	133 (25.3)	136 (27.4)	
Current	112 (20.2)	104 (19.7)	111 (22.3)	128 (23.0)	
BMI (kg/m^2^)	29.9 (0.4)	28.9 (0.4)	28.6 (0.4)	30.1 (0.4)	0.949
eGFR (mL/min)	109.0 (0.8)	99.3 (0.9)	91.1 (1.0)	84.2 (1.1)	< 0.001
Diabetes	22 (3.2)	38 (6.4)	77 (12.7)	137 (20.4)	
CVD (%)	5 (0.7)	20 (4.7)	44 (7.2)	79 (13.6)	< 0.001
Stroke	1 (0.2)	5 (1.6)	19 (3.7)	26 (4.4)	0.008

Data are expressed as numbers (weighted proportions) for categorical variables and as weighted means (Standard Error (SE)) for continuous variables. *p*-values for trend in participants’ features were computed using weighted linear and logistic regression for continuous and categorical variables, respectively

*BMI* body mass index; *CVD* cardiovascular disease; *HbA1c* hemoglobin A1c; *HDL* high density lipoprotein

### Association between sNfL levels and all-cause mortality

**Fig. 3:**
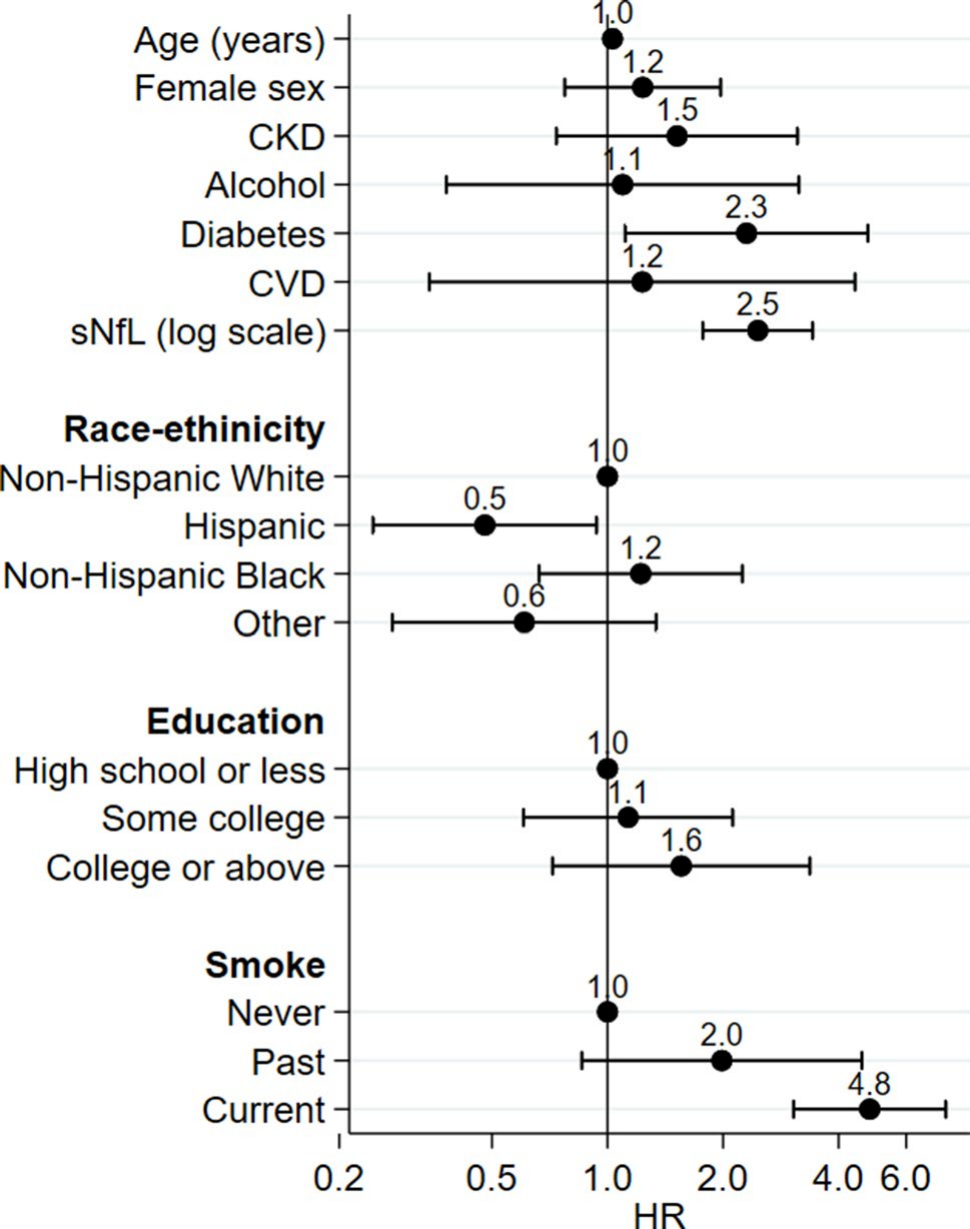
Multivariable Cox proportional hazard model evaluating the association between log-transformed serum neurofilament light chain (sNfL) levels and all-cause mortality in the studied population

Over a median follow-up of 6.1 years, 85 participants (4.1%) died. Results from the multivariable Cox proportional hazard model evaluating the association between log-transformed serum neurofilament light chain (sNfL) levels and all-cause mortality are shown in Fig. 3. Significant predictors of mortality included age, presence of diabetes, cigarette smoke and higher sNfL levels (HR per unit increase in log-transformed sNfL: 2.46, 95% CI 1.77–3.43, *p* < 0.001).

We then performed subgroup-sensitivity analyses to evaluate the consistency of our findings. As shown in Table 2, higher levels were associated with increased incidence of all-cause mortality after adjustment for potential confounders independently of age, sex, presence of obesity, eGFR, race-ethnicity and cigarette smoke. The highest HRs were identified in males and non-obese participants, as well as in participants with a reduced kidney function.

**Table 2:** Multivariable Cox proportional hazard model evaluating the association between log-transformed serum neurofilament light chain (sNfL) levels and all-cause mortality through December 2019 in specific subgroups of the studied population

Population	All-cause mortality		
	HR	95% CI	*p*-Value
Age			
< 60 years	2.57	1.65–4.01	< 0.001
≥ 60 years	2.67	1.66–4.32	0.001
Sex			
Males	3.05	1.71–5.44	0.001
Females	1.89	1.06–3.39	0.034
Renal function			
eGFR < 90 mL/min	3.02	1.66–5.50	0.001
eGFR ≥ 90 mL/min	2.38	1.35–4.18	0.005
Adiposity			
BMI < 30 kg/m^2^	3.52	1.91–6.50	0.001
BMI ≥ 30 kg/m^2^	1.93	1.30–2.88	0.003
Race-ethnicity			
Non-Hispanic white	2.51	1.60–3.96	0.001
Other ethnicities	2.01	1.44–2.82	< 0.001
Cigarette smoke			
Current smokers	2.63	1.18–5.87	0.022
Never/former smokers	2.61	1.78–3.84	< 0.001

Hazard ratios (HR) for all-cause mortality and related confidence intervals (CI) for a unit increase of log-transformed sNfL were adjusted for age, sex, race-ethnicity, chronic kidney disease, alcohol consumption, diabetes, cardiovascular disease, educational status and cigarette smoke

## Discussion

In the present study, performed on a large and representative sample of the multiethnic United States population, we made a series of observations. First, we showed that sNfL levels are independently associated with cognitive performance (as estimated by three well-validated tests) in a community setting, showing a significant, indirect relationship, which persisted after adjustment for age, sex, BMI and education. Second, we showed that after adjustment for potential confounders, higher sNfL levels were associated with an increased risk of all-cause mortality. Finally, mortality results were robust when analyses were stratified according to age, sex, BMI and kidney function.

Our results are in agreement with three recent studies evaluating the relationship between sNfL levels and mortality in a general population setting [9, 11, 22]. These previous studies, which consistently showed a detrimental effect of increased sNfL levels on mortality, had a smaller sample size and were mainly focused on middle-aged or elderly individuals or on specific ethnic groups. In contrast, mean age in the current study was 46.9 years (with 73.8% of participants being younger than 60 years). In this sense, our analysis extends previous findings to individuals of a wide age range and of different ethnic backgrounds. Moreover, given the higher number of studied individuals, we were able to perform subgroup analysis that confirmed the identified situation in all considered strata. The diverse ethnic background evaluated in the present analysis and the robust association between sNfL levels and mortality in both non-Hispanic whites and other participants suggests the possible application of sNfL measurements in all ethnicities as biomarkers of neuronal damage, without concerns of potential differences across ethnic groups.

The underlying mechanisms linking sNfL levels to mortality are still unclear. Previous studies hypothesized a role of deterioration of the nervous system to mortality [9]. In the current study, due to lack of a standardized neurological exam, history and imaging techniques, we could not evaluate whether the increased sNfL levels were due to neurological disorders. Nonetheless, the significant association between sNfL levels and cognitive performance tests, even after adjustment for age, sex, BMI and educational status, suggests that sNfL levels might be able to detect subtle neuronal degeneration as the driver of increased mortality.

In particular, the negative association between sNfL levels and performance on cognitive function tests is likely to reflect neuronal damage at the level of the central nervous system. This might imply that the use of sNfL in a general medicine setting could help identifying patients that may display subtle neuronal damage and may need referral to neurologists or clinical psychologists for formal cognitive testing. Nonetheless, more evidence in this sense should be obtained before translating our results into clinical practice.

It should be noted that the NHANES purpose is to collect a sample that is representative of the general US population. In this setting, the number of participants with clinically evident neurological disorders is likely to be low. It is, however, possible that undiagnosed subtle central or peripheral nervous system disorders mediate, completely or to a certain extent, the identified association.

Subgroup analyses identified a stronger association between sNfL levels and mortality in participants with a reduced kidney function and lower BMI. It is well known that eGFR is inversely associated with sNfL levels in people without neurological disorders [23]. As reduced kidney function might reduce the ability of sNfL to detect neuronal damage, our results are in part unexpected. A possibility is related to the fact that the causes of death may be different between patients with and without CKD as patients with CKD are at increased risk of both renal and cardiovascular events (which may be better captured by sNfL compared with other causes of death including cancer). Unfortunately, the relatively low number of events prevents us from evaluating cause-specific mortality. We also found a higher relative risk of death with increasing sNfL levels observed in non-obese compared with obese participants. Given that previous studies showed that sNfL levels are inversely related to BMI or waist-to-hip ratio [24, 25], it is possible that this biomarker has a lower ability to detect neurological diseases in this patients’ population, where false negative results may occur.

Our study has several strengths. It is a large study performed in an unselected sample of US adults, including both sexes and participants of different ethnic background. The high number of participants included yielded enough statistical power to perform sensitivity analyses and evaluate the impact of several predictors in multivariable models. Being based on NHANES data, our results have a high degree of external validity as the purpose of the survey is to be representative of the overall US population. Acquisition of clinical, laboratory and anthropometric data was standardized and homogenous and sNfL levels were measured with a highly reproducible method. Finally, we focused on hard clinical outcomes with very high degree of retention of participants, as data were based on the National Death Index.

On the same lines, several limitations should also be acknowledged.

First, given the relatively low number of events, we could only provide evidence on all-cause mortality, while data on non-fatal cardiovascular or neurological events were not available. Second, while cognitive function tests were performed in a subgroup of subjects, the cohort has not been thoroughly studied from a neurological point of view, both in terms of physical findings and imaging techniques. Related to this point, no objective measurement of sensorimotor neuropathy was available in the dataset, preventing us from evaluating to which degree neuropathy rather than central nervous system conditions might account for the observed associations. Finally, despite adjustment for several variables, the possibility of residual confounding cannot be completely excluded.

## Conclusions

In conclusion, in this large, population-based cohort study, we show that sNfL levels are strongly associated with cognitive performance (as estimated by three well-validated tests) in a community setting. Moreover, higher sNfL levels were associated with an increased risk of all-cause mortality in the population as a whole, as well as when analyses were stratified according to age, sex, BMI and kidney function. Further large-scale and prospective studies with more in-depth characterization of nervous system structure and function are needed to replicate our results and evaluate whether mechanisms different from neurodegeneration might account for this association.

## Data Availability

All data analyzed during this study are publicly available at the NHANES website.

## Code availability

Code is available from the corresponding author upon reasonable request.
